# Amyloid pathology and axonal injury after brain trauma

**DOI:** 10.1212/WNL.0000000000002413

**Published:** 2016-03-01

**Authors:** Gregory Scott, Anil F. Ramlackhansingh, Paul Edison, Peter Hellyer, James Cole, Mattia Veronese, Rob Leech, Richard J. Greenwood, Federico E. Turkheimer, Steve M. Gentleman, Rolf A. Heckemann, Paul M. Matthews, David J. Brooks, David J. Sharp

**Affiliations:** From the Division of Brain Sciences (G.S., A.F.R., P.E., P.H., J.C., R.L., S.M.G., R.A.H., P.M.M., D.J.B., D.J.S.), Department of Medicine, Imperial College London; Institute of Psychiatry, Psychology & Neuroscience (P.H., M.V., F.E.T.), King's College London; Institute of Neurology (R.J.G.), University College London, UK; MedTech West at Sahlgrenska University Hospital (R.A.H.), University of Gothenburg, Sweden; and Institute of Clinical Medicine (D.J.B.), Aarhus University, Denmark.

## Abstract

**Objective:**

To image β-amyloid (Aβ) plaque burden in long-term survivors of traumatic brain injury (TBI), test whether traumatic axonal injury and Aβ are correlated, and compare the spatial distribution of Aβ to Alzheimer disease (AD).

**Methods:**

Patients 11 months to 17 years after moderate–severe TBI underwent ^11^C-Pittsburgh compound B (^11^C-PiB)-PET, structural and diffusion MRI, and neuropsychological examination. Healthy aged controls and patients with AD underwent PET and structural MRI. Binding potential (BP_ND_) images of ^11^C-PiB, which index Aβ plaque density, were computed using an automatic reference region extraction procedure. Voxelwise and regional differences in BP_ND_ were assessed. In TBI, a measure of white matter integrity, fractional anisotropy, was estimated and correlated with ^11^C-PiB BP_ND._

**Results:**

Twenty-eight participants (9 with TBI, 9 controls, 10 with AD) were assessed. Increased ^11^C-PiB BP_ND_ was found in TBI vs controls in the posterior cingulate cortex and cerebellum. Binding in the posterior cingulate cortex increased with decreasing fractional anisotropy of associated white matter tracts and increased with time since injury. Compared to AD, binding after TBI was lower in neocortical regions but increased in the cerebellum.

**Conclusions:**

Increased Aβ burden was observed in TBI. The distribution overlaps with, but is distinct from, that of AD. This suggests a mechanistic link between TBI and the development of neuropathologic features of dementia, which may relate to axonal damage produced by the injury.

Traumatic brain injury (TBI) is the leading cause of disability in young adults.^1^ Survivors may deteriorate clinically many years after injury,^2^ and TBI is thought to be a major risk factor for dementia.^3^ However, the mechanisms relating acute injury to later neurodegeneration are unclear, and the prevalence of distinct types of dementia such as Alzheimer disease (AD) and chronic traumatic encephalopathy is uncertain.^3^

A mechanistic link between moderate to severe TBI and AD is suggested by the observation that β-amyloid (Aβ) aggregates are found in brains of up to a third of patients who die acutely after TBI,^3^ and in a similar proportion who survive for a year or more.^4^ Traumatic axonal injury (TAI), a pathology consistently observed after TBI,^5^ offers a potential mechanism for Aβ genesis.^6^ It is postulated that abundant amyloid precursor protein, which accumulates in damaged axons, is aberrantly cleaved to form Aβ, which subsequently aggregates as Aβ plaques.^6^ Immunohistochemical evidence also shows that the enzymes necessary for Aβ cleavage accumulate at sites of TAI.^6^

Localization of fibrillar Aβ pathology in vivo is possible using PET. The amyloid tracer ^11^C-Pittsburgh compound B (^11^C-PiB) shows robust retention in brains of patients with AD^7^ in a pattern that corresponds with neuropathologic studies of Aβ plaque distribution, with increases initially in the precuneus/posterior cingulate cortex (PCC), frontal cortex, and caudate nuclei, then lateral temporal and parietal cortex.^8,9^ Recently, a pilot ^11^C-PiB-PET study in patients with moderate to severe TBI less than 1 year after injury found increased uptake in cortical gray matter (GM) and striatum.^10^ These findings suggest that Aβ imaging in the chronic phase after TBI may inform our understanding of neurodegeneration in long-term survivors of TBI.

Diffusion tensor imaging (DTI) can be used to estimate in vivo the degree of axonal injury following TBI.^11–14^ In this study, we combined ^11^C-PiB-PET and DTI to test the following hypotheses: (1) Aβ pathology is present in long-term survivors of TBI without dementia; and (2) Aβ pathology after moderate to severe TBI is related to the amount and distribution of TAI.

## METHODS

### Study design and participants.

In this cross-sectional study, 9 patients with a history of a single moderate–severe TBI based on Mayo criteria^15^ were assessed with ^11^C-PiB-PET, structural T1 MRI, DTI, and neuropsychological examination. Patients were recruited at least 11 months after their injury (e-Methods on the *Neurology*® Web site at Neurology.org). For comparison of ^11^C-PiB binding, a group of patients with AD had ^11^C-PiB-PET and structural MRI (e-Methods). We used 3 healthy control groups: (1) for comparison of ^11^C-PiB binding, a group of healthy aged controls had PiB-PET and structural MRI; (2) for comparison of neuropsychological performance, a second group of healthy controls, age-matched to the patients with TBI, underwent neuropsychological assessment; and (3) for comparison of white matter (WM) integrity, a third group of healthy aged-matched controls underwent structural MRI and DTI.

### Standard protocol approvals, registrations, and patient consents.

The project was approved by Hammersmith and Queen Charlotte's and Chelsea Research Ethics Committee. All participants gave written informed consent.

### Procedures.

A neuropsychological test battery was performed on patients with TBI and age-matched controls (e-Methods). Patients with AD and healthy aged controls underwent the Mini-Mental State Examination.

An overview of the imaging methods is shown in figure e-1. All patients and healthy aged controls had ^11^C-PiB-PET using a Siemens ECAT EXACT HR+ scanner (Siemens Medical Systems, Erlangen, Germany). ^11^C-PiB was manufactured and supplied by Hammersmith Imanet (London, UK). All participants had an IV bolus injection of ^11^C-PiB, mean dose 370 MBq, and dynamic PET emission scans were acquired over 90 minutes.

To generate nondisplaceable binding potential (BP_ND_) images of ^11^C-PiB, we used a supervised clustering procedure for automatic reference region extraction.^16^ T1 images were automatically segmented into GM and WM. The tissue segmentations were warped to an average group template image using a diffeomorphic nonlinear image registration procedure (DARTEL).^17^ The group template image was then registered to Montreal Neurological Institute (MNI) space. Each individual's ^11^C-PiB BP_ND_ image was coregistered to their T1 image, then the individual flow-fields and template registration obtained from the DARTEL procedure were used to warp the BP_ND_ images to MNI space. The normalized BP_ND_ images were masked using the thresholded GM template and smoothed (8 mm full width at half maximum) (e-Methods).

^11^C-PiB binding potentials were also sampled from anatomically defined regions of interest (ROIs). The MAPER (multi-atlas propagation with enhanced registration) procedure was used to generate native-space ROIs.^18^ To improve sampling accuracy, ROI masks were intersected with thresholded tissue probability maps (e-Methods). To confirm that the hippocampal ROI results were not an effect of mislabeling due to atrophy, sampling was repeated on hippocampal masks that were manually segmented using a harmonized protocol.^19^

In patients with focal injuries, lesions apparent on T1 imaging were manually segmented and excluded from ROI and voxelwise analyses. We also investigated ^11^C-PiB binding within a lesion, the lesion penumbra, and normal-appearing GM in the same hemisphere (e-Methods).

Patients with TBI and a group of healthy aged-matched controls underwent DTI (e-Methods). Voxelwise maps of fractional anisotropy (FA), a measure of WM tract integrity after TBI, were calculated using the FSL Diffusion Toolkit.^20^ The FA maps were skeletonized using tract-based spatial statistics (TBSS).^21^ We calculated the mean FA of the TBSS skeleton and also of selected tracts from the Johns Hopkins University WM Tractography Atlas.^22^ We chose tracts connected to GM regions that had shown increased ^11^C-PiB binding in TBI. We also sampled the corticospinal tract as a control, since this was not connected to these regions.

### Statistical analysis.

Group differences in neuropsychological measures were examined using independent sample *t* tests and Mann–Whitney *U* tests in SPSS version 21 (IBM Corp., Armonk, NY). Voxelwise differences in BP_ND_ between groups were assessed using nonparametric permutation tests in FSL with 10,000 permutations. This approach incorporated a tool that uses voxelwise regressors to exclude individual lesions from the analysis.^23^ Results were cluster-corrected using threshold-free cluster enhancement and a family-wise error rate of <0.05. For presentation, images were thresholded at *p* < 0.001 uncorrected. For ROI analysis, regional BP_ND_ was compared using repeated-measures analysis of variance (ANOVA), in SPSS. Mean FA values of WM tracts were compared between patients with TBI and controls using unpaired 2-sample *t* tests. Regional ^11^C-PiB was correlated with mean FA values, age, time since injury, and neuropsychological test scores (e-Methods). Mean FA values were correlated with age and time since injury. To correct for multiple comparisons, a false discovery rate threshold was calculated using *q* = 0.05.

## RESULTS

Nine patients with TBI (mean age 44.1 ± 4.9 years, range 38–54) were recruited 11 months to 17 years after injury (table 1). Ten patients with AD (mean age 67.3 ± 4.5, range 58–76) and 9 healthy aged controls (62.3 ± 4.3, range 55–66) were also assessed. In addition, a group of 15 age-matched controls (37.3 ± 11.3, range 19–60) underwent neuropsychological assessment and a separate group of 11 age-matched controls (40.9 ± 5.4, range 35–51) underwent MRI and DTI. None of the patients had a clinical diagnosis of posttraumatic stress disorder or anxiety disorder. One patient had a diagnosis of depression following the TBI. Structural T1 scans were reviewed by a senior neuroradiologist. Four patients with TBI had no abnormalities. The remaining 5 had focal lesions, with damage in the frontal (n = 3) or temporal (n = 3) lobes (figure e-2). One patient had undergone a parietotemporal lobectomy following TBI.

**Table 1:**
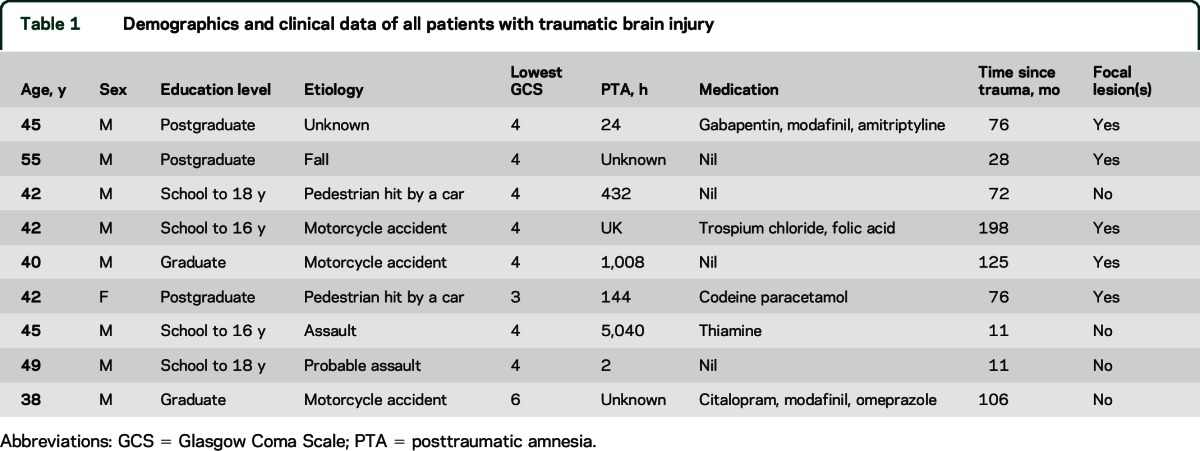
Demographics and clinical data of all patients with traumatic brain injury

### Neuropsychological impairment after TBI.

The patients with TBI showed impairments in neuropsychological performance compared to age-matched healthy controls. Significantly poorer responses were seen across a range of tasks, including tests of attention, information processing speed, and cognitive flexibility (table e-1). In other tests, the patients were well matched with controls. As expected, the AD group had a lower Mini-Mental State Examination score (mean 21.1/30 ± 4.1) than healthy aged controls (all 30/30, *t* = −6.54, *df* = 9, *p* < 0.001).

### Amyloid pathology after TBI is detected by ^11^C-PiB binding.

**Figure 1:**
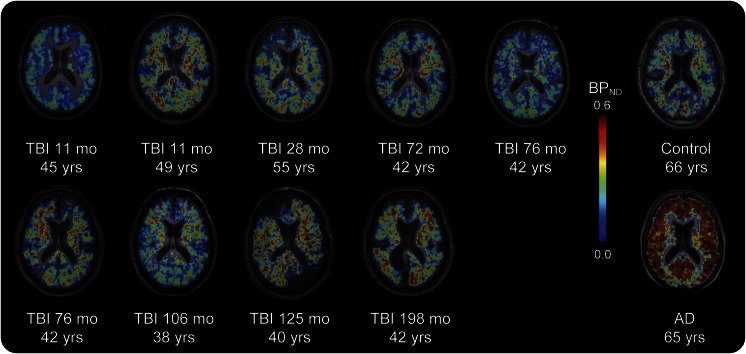
^11^C-PiB binding following TBI Images of axial T1 MRI are superimposed with ^11^C-PiB BP_ND_ maps for all patients with TBI and a representative healthy aged control and a participant with AD. For patients with TBI, the interval in months from the time of TBI to PET scanning and the age in years of each participant at scanning is also shown. AD = Alzheimer disease; BP_ND_ = nondisplaceable binding potential; ^11^C-PiB = ^11^C-Pittsburgh compound B; TBI = traumatic brain injury.

**Figure 2:**
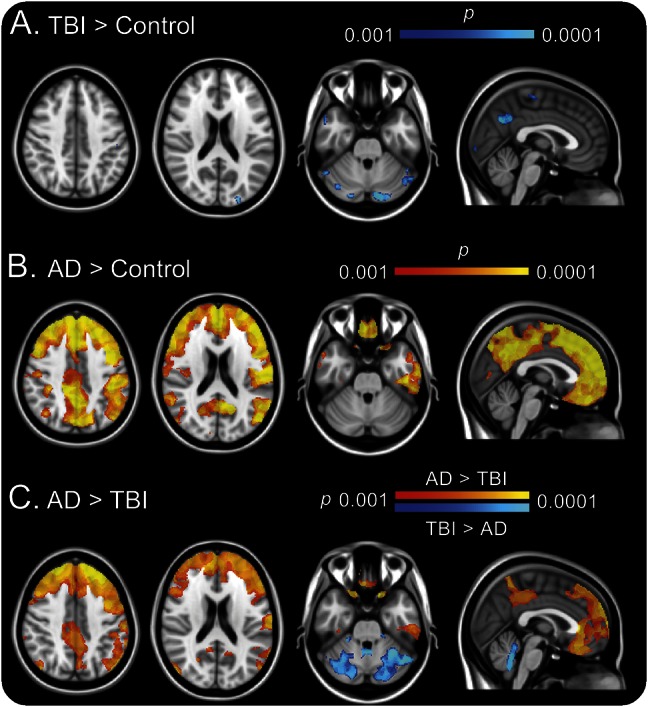
Increased ^11^C-PiB binding in TBI and AD (A) Blue–light blue areas showed significantly increased ^11^C-PiB BP_ND_ in TBI compared to healthy aged controls. (B) Red–yellow areas showed significantly increased binding in AD compared to controls. (C) Blue–light blue areas showed significantly increased ^11^C-PiB BP_ND_ in TBI compared to AD. Red–yellow areas showed significantly increased binding in AD compared to TBI. Images are shown thresholded at *p* < 0.001 uncorrected. AD = Alzheimer disease; BP_ND_ = nondisplaceable binding potential; ^11^C-PiB = ^11^C-Pittsburgh compound B; TBI = traumatic brain injury.

**Figure 3:**
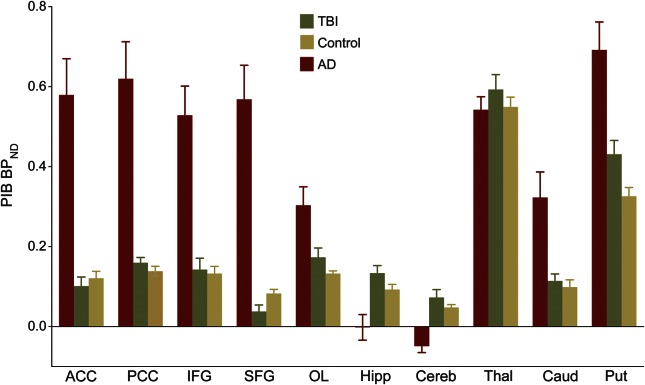
^11^C-PiB BP_ND_ region of interest analysis Mean group ^11^C-PiB BP_ND_ ± SEM is shown for patients with TBI (green), patients with AD (red), and healthy aged controls (yellow). ACC = anterior cingulate cortex; AD = Alzheimer disease; BP_ND_ = nondisplaceable binding potential; ^11^C-PiB = ^11^C-Pittsburgh compound B; Caud = caudate; Cereb = cerebellum; Hipp = hippocampus; IFG = inferior frontal gyrus; OL = occipital lobe; PCC = posterior cingulate cortex; Put = putamen; SFG = superior frontal gyrus; Skel = skeleton; TBI = traumatic brain injury; Thal = thalamus.

^11^C-PiB BP_ND_ images of the TBI group are shown for individual patients (figure 1). Slices from a representative patient with AD and a healthy aged control are shown. Direct comparison of patients with TBI and healthy aged controls showed areas of increased ^11^C-PiB BP_ND_ following TBI (figure 2A). Peaks of increased ^11^C-PiB BP_ND_ corrected for multiple comparisons were observed in the precuneus/PCC and cerebellum. There were no areas of decreased binding in patients with TBI compared to controls. We performed a confirmatory ROI analysis using anatomically defined regions (figure 3). ANOVA of BP_ND_ sampled from 10 ROIs in the TBI and healthy control groups showed a significant group-by-region interaction (*F*_3.127, 50.036_ = 2.984, *p* = 0.038, Greenhouse-Geisser correction applied). The partial η^2^ effect size estimate was 0.157. The interaction was driven by increased binding in the putamen of patients with TBI (*t* = 2.573, *df* = 16, *p* = 0.020) and a decrease in the superior frontal gyrus (*t* = −2.312, *df* = 16, *p* = 0.034), but nonsignificant differences elsewhere.

### ^11^C-PiB binding is decreased around focal lesions.

Visual inspection of individual TBI BP_ND_ images showed no binding in the vicinity of focal cortical lesions evident on structural MRI. To confirm this, we sampled binding in ROIs placed in and around the most prominent lesion in each brain. As expected, there was no specific binding in the focal lesion. In addition, binding in the penumbra was reduced compared to normal-appearing GM in the same hemisphere (*t* = −11.54, *df* = 4, *p* < 0.001).

### ^11^C-PiB binding after TBI is correlated with WM damage and time since injury.

**Figure 4:**
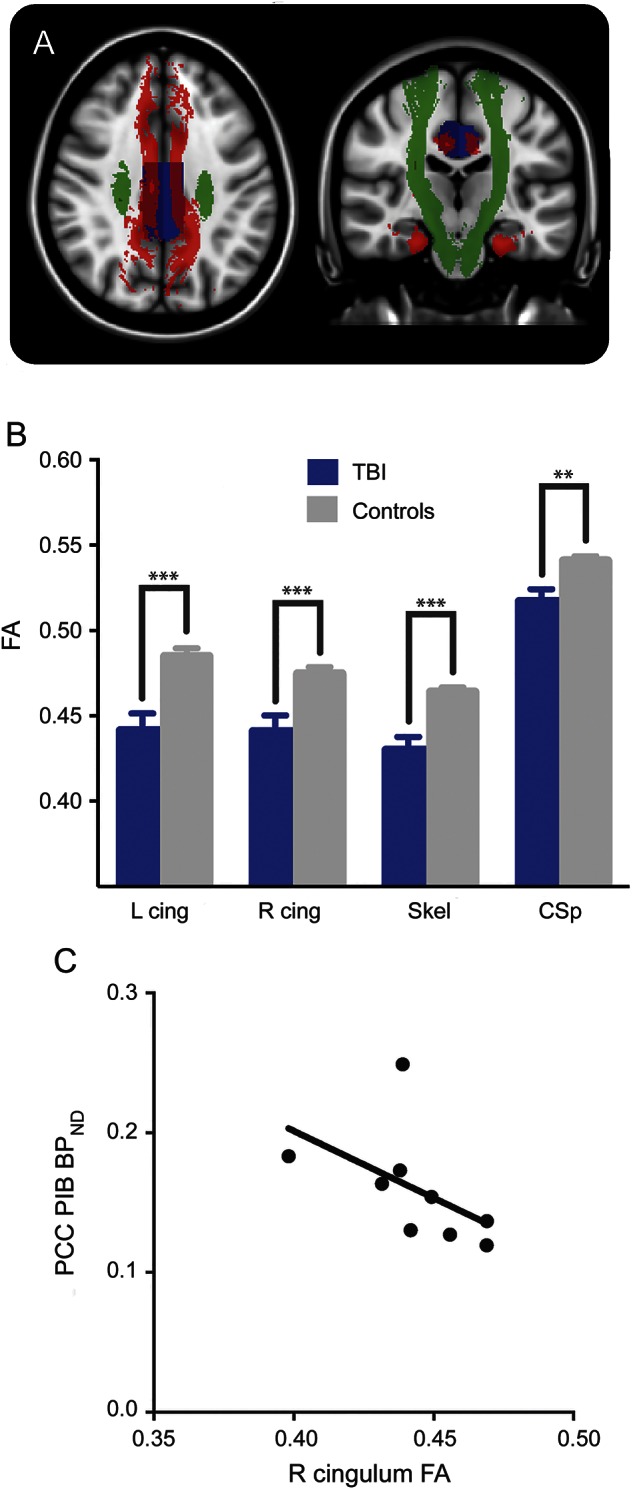
Relationship between white matter damage and regional ^11^C-PiB BP_ND_ in patients with TBI (A) Selected white matter tracts from the Johns Hopkins University tractography atlas and region of interest from the MAPER (multi-atlas propagation with enhanced registration) segmentation are shown on an MNI152 standard image. The tracts in red are the left and right cingulum–cingulate bundle combined with left and right cingulum–hippocampus tract. The regional segmentation of the PCC is shown (blue), which receives connections from these tracts. The corticospinal tract (green) is not connected to the PCC. FA, a measure of white matter integrity, was sampled from the tracts in patients with TBI using diffusion tensor imaging and related to regional ^11^C-PiB BP_ND_ sampled in the PCC. (B) The mean FA of all tracts tested was reduced in TBI (blue) compared to controls (gray) (**p < 0.01, ***p < 0.001). (C) ^11^C-PiB BP_ND_ in the PCC increased with decreasing FA in the right cingulum. BP_ND_ = nondisplaceable binding potential; ^11^C-PiB = ^11^C-Pittsburgh compound B; cing = cingulum; CSp = corticospinal tract; FA = fractional anisotropy; PCC = posterior cingulate cortex; TBI = traumatic brain injury.

We next examined whether Aβ plaque pathology in the PCC was associated with the degree of TAI in the patients with TBI. We tested the hypothesis that regional GM ^11^C-PiB binding increases with lower FA (indicative of axonal injury) in the cingulum bundles that were directly connected to the PCC (figure 4A). Mean FA in all tracts examined was reduced as expected (figure 4B). PCC BP_ND_ was negatively correlated in both the left cingulum (*R* = −0.733, *p* = 0.031) and right cingulum (*R* = −0.750, *p* = 0.025, figure 4C), a relationship that survived correction for the age of the patient (*R* = −0.758, *p* = 0.029; *R* = −0.787, *p* = 0.020). The mean FA of the WM skeleton also showed a correlation with PCC binding (*R* = −0.733, *p* = 0.031), although this was only of borderline significance when correcting for age (*R* = −0.694, *p* = 0.056). There was no significant correlation found with the corticospinal tract FA. ^11^C-PiB binding in the PCC also increased with time since injury duration (*R* = 0.767, *p* = 0.021), although this was not significant after correcting for age (*R* = 0.625, *p* = 0.097). Of the 4 FA measures, the mean FA of the left cingulum also correlated with time since injury (*R* = −0.717, *p* = 0.037). There was no independent relationship between ^11^C-PiB binding and FA after correction for time since injury. There was also no correlation between patient age and ^11^C-PiB binding or FA.

### ^11^C-PiB binding is not correlated with neuropsychological impairment in TBI.

There were no significant correlations between PCC binding and behavioral performance in the patients with TBI.

### Distinct distributions of ^11^C-PiB binding in TBI and AD.

The direct contrast of AD and controls showed increased ^11^C-PiB binding in AD association cortex and cingulate (figure 2B). Conjunction analysis showed that ^11^C-PiB binding was increased in a cluster within the precuneus/PCC in both AD and TBI compared to controls. In general, ^11^C-PiB binding was higher in AD than TBI across regions, but the TBI cases had relatively increased binding in the cerebellum (figure 2C). Interrogating ROI data with ANOVA confirmed the voxel-level findings. Increased ^11^C-PiB binding was seen in cortical association and cingulate regions in AD whereas increased cerebellar binding was seen in TBI (e-Results). There was no correlation between patient age and regional ^11^C-PiB binding within any of the 3 participant groups.

## DISCUSSION

TBI can predispose patients to various types of dementia, but there is no consensus about how post-TBI dementia syndromes should be classified or diagnosed. Patients often clinically deteriorate years after TBI,^2^ but it is difficult to determine whether this is related to the prior head injury. Improved methods of characterizing neurodegenerative processes triggered by TBI are needed. We investigated amyloid pathology using ^11^C-PiB-PET. For the first time, we show in vivo that increases in ^11^C-PiB binding are present in long-term survivors of TBI in a distribution overlapping with AD but also involving the cerebellum.^24^ A mechanistic link between axonal injury and amyloid pathology is suggested by the relationship between cortical ^11^C-PiB binding and WM damage in connected tracts.

In AD, Aβ deposition usually begins in inferior frontal and cingulate association cortex, extending into other association cortical regions. Early deposition is seen in the PCC,^25^ and we observed increased ^11^C-PiB uptake in both patients with TBI and those with AD. While the ventromedial frontal cortex is affected early in AD, the hippocampus and cerebellum are not usually involved until much later in the disease.^24^ In keeping with this pattern, we observed strong ^11^C-PiB binding in the prefrontal cortex in our patients with AD, but relatively low levels in the hippocampus and cerebellum. However, a different pattern was observed in our patients with TBI, who had increased cerebellar ^11^C-PiB binding relative to both AD and controls. The distinct distribution of ^11^C-PiB binding in the 2 contexts suggests that amyloid pathology is triggered by a different mechanism after TBI, which is likely to relate to biomechanical forces underlying the distinctive pattern of Aβ plaque pathology seen in cases of chronic traumatic encephalopathy.^26^ TBI might also accelerate an aging process^27^ and our results may reflect this change in aging trajectory, particularly considering that the increased ^11^C-PiB binding after TBI was observed in comparison to a much older aged control group. However, in keeping with studies of AD,^8^ ^11^C-PiB binding did not correlate with cognitive impairment.

Axonal damage produced at the time of injury may act as an initial trigger for Aβ production and accumulation of amyloid pathology.^6^ In keeping with this possibility, we observed an association between the extent of WM damage and ^11^C-PiB binding in the PCC following TBI. The biomechanical effects of torsional and shear stress on WM tracts produce TAI, and this is thought to be an important factor driving overproduction of Aβ, leading to its aggregation in the acute phase.^3^ Axons and their surrounding myelin are damaged, and the pathologic effects of injury remain visible for many years, particularly in long-distance WM tracts.^28^ Animal models and human autopsy studies provide evidence that Aβ is produced at the site of axonal injury shortly after TBI.^6^

The relationship between ^11^C-PiB binding and WM damage was seen in the cingulum bundles, which connect to the PCC. The relationship was not observed in the corticospinal tract, which is not directly connected to the PCC, suggesting a more specific link between the 2 observations. Misfolded proteins, including Aβ, have the capacity to move from neuron to neuron via prion-like transsynaptic spread,^29,30^ and computational simulations show that a simple diffusion mechanism can produce the complex patterns of brain atrophy observed in AD if large-scale WM structure is factored into the model.^31^ The implication for TBI is that the WM may be both a source of Aβ and a conduit for Aβ diffusion. The correlation between measures of TAI and Aβ pathology in the PCC may reflect its role as a highly connected cortical hub,^32^ which integrates damage that spreads from damaged WM tracts. The time elapsed since a patient's injury also correlated with ^11^C-PiB binding, suggesting there is a progressive neurodegenerative process. Our results suggest that ^11^C-PiB binding, WM structure, age, and time since injury are interrelated, and longitudinal studies with larger numbers will be needed to clarify the causal relationships. Such studies should also examine ^11^C-PiB binding in the context of host genotype, particularly *APOE*,^33^ which was not addressed here.

Our findings are broadly consistent with a previous ^11^C-PiB study in patients with TBI scanned less than 1 year after injury (median 11 days). Hong et al.^10^ showed increased cortical and striatal ^11^C-PiB binding early after TBI. Of note, the validity of in vivo neuroimaging was supported by [^3^H]PiB autoradiography and Aβ immunohistochemistry. In contrast to our study, this earlier work used the cerebellum as a reference region for quantification of ^11^C-PiB binding, assuming that there was minimal Aβ plaque density in the cerebellum and that the ratio of cortical to cerebellar binding provided a measure of cortical Aβ burden.^34^ Hong et al. provide evidence to support this assumption early after TBI. However, our results demonstrate that this is not the case in the chronic phase after TBI. Our initial analyses in TBI using the cerebellum as a reference region suggested decreased cortical ^11^C-PiB binding. Therefore, we used a procedure for automatic reference region extraction that has been validated in familial AD and does not require a single anatomically defined reference region.^16^

Our study has a number of potential limitations. First, given the small sample size, our findings should be regarded as preliminary. Second, the ^11^C-PiB healthy controls were age-matched to the AD group, and so were older than the TBI group. Although 2 separate age-matched control groups would have been preferable, Aβ pathology increases with age^35^ and so a comparison with older healthy controls is likely to have reduced our sensitivity to detect a relative increase in the younger TBI group. Therefore, the presence of abnormalities in a relatively young TBI group is even more striking. Third, it is possible that GM tissue differences such as atrophy, associated with AD or aging, could have biased our group contrast results. A number of analysis steps were used to minimize this possibility: an advanced algorithm for optimized registration of brain images into standard space (DARTEL)^36^; ^11^C-PiB binding was only assessed in regions where the GM probability was high; and ROI analyses, based on both automated segmentations, were used to provide confirmatory results. To control for the possible effects of focal injury after TBI, we also excluded lesioned areas from the analysis. It is possible that the extent of focal lesions was underestimated as we used T1 imaging to segment the lesions. However, since ^11^C-PiB binding was reduced in visible lesions, this possibility would have biased the analysis against detecting increases in ^11^C-PiB.

We provide ^11^C-PiB-PET evidence for the presence of amyloid pathology many years after injury in patients with TBI without dementia. The distribution of ^11^C-PiB binding partially overlapped with that seen in typical AD but also affected the cerebellum, unlike in AD. This suggests a different mechanism for amyloid plaque genesis. Our findings support the hypothesis that amyloid plaque pathology is related to the presence of axonal damage produced subsequent to the TBI.

## Supplementary Material

Data Supplement

Accompanying Editorial

## GLOSSARY

Aβ: β-amyloid
AD: Alzheimer disease
ANOVA: analysis of variance
BPND: nondisplaceable binding potential
^11^C-PiB: ^11^C-Pittsburgh compound B
DTI: diffusion tensor imaging
FA: fractional anisotropy
GM: gray matter
MNI: Montreal Neurological Institute
PCC: posterior cingulate cortex
ROI: region of interest
TAI: traumatic axonal injury
TBI: traumatic brain injury
TBSS: tract-based spatial statistics
WM: white matter
