# Supplementary material for: Amyloid pathology and axonal injury after brain trauma
Source: Neurology. 2016 Mar 1;86(9):821–8. doi: 10.1212/WNL.0000000000002413 (PMC4793784; doi:10.1212/WNL.0000000000002413)
Supplement: Data Supplement [file supp_WNL.0000000000002413_Figure_e-1.pdf]

Figure e-1. Overview of 11C-PIB imaging analysis.

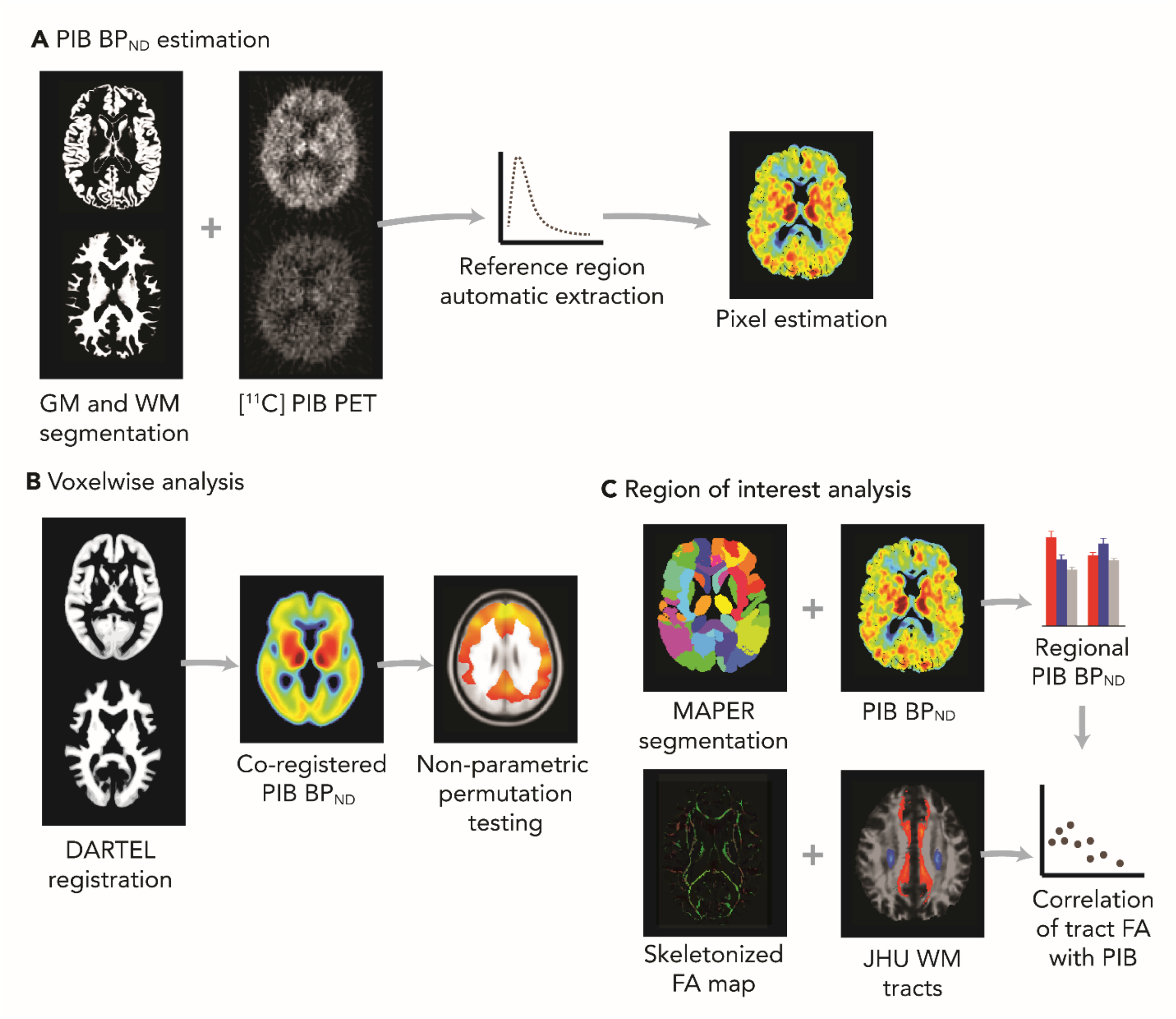

(A) Binding potential (BPND) images of 11C-PIB, proportional to amyloid-β plaque density, were estimated from dynamic 11C-PIB PET data with an automatic reference region extraction procedure, using grey matter (GM) and white matter (WM) segmentations from T1 MRI to define tissue classes. (B) GM and WM segmentations were warped to a group template image using a diffeomorphic non-linear image registration procedure (DARTEL). The same normalisation was applied to 11C-PIB BPND images. Voxelwise differences in BPND between groups were assessed using non-parametric permutation tests. (C) Native space anatomical regions of interest (ROIs) for sampling BPND were generated using the MAPER (multi-atlas propagation with enhanced registration) procedure. In TBI patients, fractional anisotropy (FA), a measure of white matter integrity, was sampled from the skeletonized white matter tracts defined by the Johns Hopkins University white matter tractography atlas, and related to regional 11C-PIB binding.
