# Supplementary material for: Amyloid pathology and axonal injury after brain trauma
Source: Neurology. 2016 Mar 1;86(9):821–8. doi: 10.1212/WNL.0000000000002413 (PMC4793784; doi:10.1212/WNL.0000000000002413)
Supplement: Data Supplement [file supp_WNL.0000000000002413_Figure_e-2.pdf]

Figure e-2. Lesion map in traumatic brain injury patients.

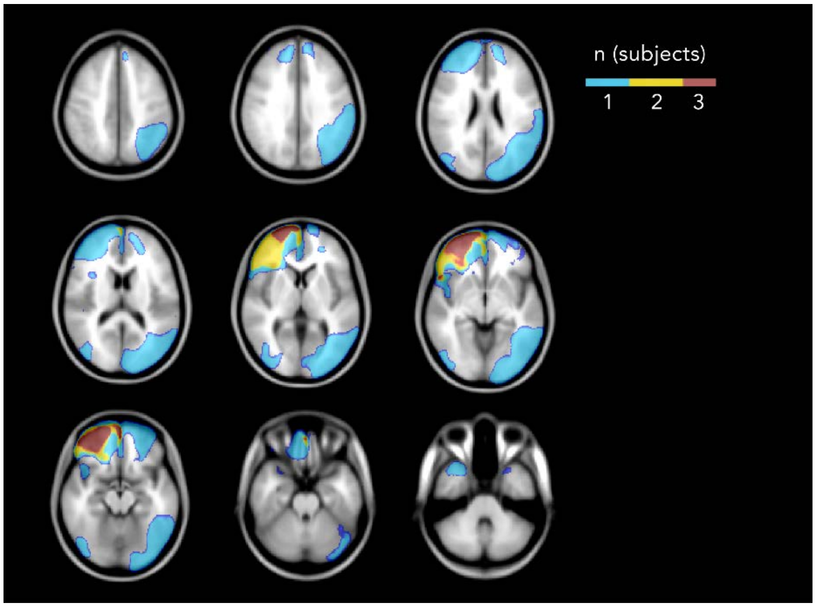

Colored regions indicate regions affected by lesions in one or more patients. Maps were formed by summation of the co-registered lesion masks of individual patients. Voxels corresponding to lesions were removed from subsequent analyses (see main text).
