# Supplementary material for: Amyloid pathology and axonal injury after brain trauma
Source: Neurology. 2016 Mar 1;86(9):821–8. doi: 10.1212/WNL.0000000000002413 (PMC4793784; doi:10.1212/WNL.0000000000002413)
Supplement: Data Supplement [file supp_WNL.0000000000002413_Supplemental_Material.pdf]

Supplementary Methods

Study design and participants

Traumatic brain injury (TBI) patients were recruited at least 11 months after their injury from specialist TBI clinics. Eligible patients had a history of a single moderate-severe TBI based on Mayo criteria[1], without pre-existing psychiatric or neurological illness, a history of other significant TBI, current or previous drug or alcohol abuse, pregnancy or breast feeding, or contraindications to MRI.

For comparison of ^{11}C-PIB binding, a group of patients with a diagnosis of clinically probable idiopathic AD based on NINDS-ADRDA criteria[2] had ^{11}C-PIB PET and structural MRI. AD patients were recruited from specialist dementia clinics at the Hammersmith Hospitals Trust and the National Hospital for Neurology and Neurosurgery, London, UK.

Data were collected between 2006 and 2010. Controls were recruited by local advertisement and from spouses of patients. We derived the study sample size on the basis of earlier PET studies that had explored PIB binding in AD patients and control subjects (e.g. [3]).

Procedures

Neuropsychological assessment

TBI patients and a group of age-matched controls underwent a neuropsychological test battery. The test battery was designed to assess domains of cognitive function often observed to be impaired following TBI. The Verbal Fluency/Letter Fluency (F + A + S) and Color-Word Interference subtests from the Delis-Kaplan Executive Function System (D-KEFS)[4] were administered to assess word generation fluency, complex information processing speed, inhibition and cognitive flexibility. The Trail Making Test (TMT)[5] was used to assess basic perceptual and complex processing speed, attentional capacity, set maintenance and set shifting. A computerised two-choice left/right (<<< or >>>) button-press choice-reaction task (CRT) was used as a simple measure of processing speed (median reaction time on correct trials). The TMT Part A, as well as the two baseline conditions of the D-KEFS Color-Word Interference test, Color Naming and Word Reading also measured simple processing speed. The Digit Span subtest of the Wechsler Memory Scale – Third Edition (WMS-III)[6] assessed initial and working memory. The Logical Memory I and II subtests of the WMS-III were used as a measure of immediate and delayed verbal recall. The People Test (PT) from the Doors and People Test battery[7] was used as a measure of associative learning and recall (immediate recall). In addition, the Wechsler Test of Adult Reading (WTAR)[8] was used to provide an estimate of pre-morbid intellectual functioning. Current verbal and nonverbal reasoning ability were assessed via the Wechsler Abbreviated Scale of Intelligence (WASI)[9] Similarities and Matrix Reasoning subtests. 15 age-matched controls (12 males, mean age 37.3±11.3 years, range 19-60 years) underwent neuropsychological assessment. Following exploratory data analysis, control subjects identified as extreme outliers (≥ 3 x interquartile range) were excluded from subsequent analyses. Group differences in neuropsychological measures were examined using independent sample t-tests, using Levene's test for equality of variances. We repeated the analyses using Mann-Whitney U tests. All tests were

performed using SPSS Version 21. The number of patient and control subjects used for each test as well as Cohen's d values[10] are provided in Table e-1.

Procedure for automatic reference region extraction

To generate non-displaceable binding potential (BP_{ND}) images of ^{11}C-PIB, a supervised clustering procedure for automatic reference region extraction was used. [^{11}C]-Pittsburgh Compound-B ([^{11}C]PIB) binding in Alzheimer's disease (AD) and healthy aging studies is usually quantified using the cerebellum as a reference region, where it is assumed that Aβ plaque density (and hence minimal specific binding) is minimal. Under this assumption, the ratio of cortical to cerebellar binding provides an accurate measure of cortical Aβ burden[11]. If there is significant Aβ plaque pathology in the cerebellum then this assumption is invalid. For example, cerebellar binding is observed in certain types of familial AD[12]. It was unclear whether cerebellar Aβ plaque pathology is present in the chronic phase, and our initial analyses using the cerebellum as a reference region suggested increased cerebellar binding (i.e. values <1 for the cortical/cerebellar ratio). For these reasons, we used a supervised clustering method[13]. This analysis approach models each gray matter voxel as the linear combination of three predefined kinetic classes (normal grey matter, lesion grey matter, and blood pool), and extracts reference voxels in which the contribution of the normal gray matter class is high. This approach has been validated in idiopathic AD and shown to be effective in familial AD, where increased cerebellar uptake has been demonstrated. The automated reference region extraction procedure and pixel estimation were carried out as described by Ikoma et al.[13] to produce individual [^{11}C]PIB BP_{ND} images in native space.

PET and T1 processing

PET and T1 MRI data were processed in SPM8. T1 images were automatically segmented into grey matter (GM) and white matter (WM). The tissue segmentations were warped to a group template image, representing the average of all participants (TBI, AD, healthy aged controls), with a diffeomorphic non-linear image registration procedure (DARTEL), and the group template image registered to Montreal Neurological Institute (MNI) space using affine registration. This procedure is a standard feature of the DARTEL implementation of SPM8, whereby the complete transformation from each individual's native T1 to MNI is achieved by applying the individual flow-fields to the T1 followed by the affine transformation from template to MNI space. Each individual's [^{11}C]PIB BP_{ND} image was registered to the T1 image using affine registration. The DARTEL transformation to MNI space was then used to warp the T1 space BP_{ND} image to MNI space.

For voxelwise statistical analysis, the normalized PET images obtained were masked so as to include only GM voxels of interest, and to prevent any overlap between voxels included in analyses with GM and those with WM. The GM mask was obtained by thresholding the GM customized template to >0.3. This approach has the benefit of reducing possible edge effects around the border between GM, WM and CSF[14-16]. The same binary mask was applied twice, before and after smoothing, to avoid contamination of misclassified voxels by smoothing in the first case, and large edge effects in the second case [15]. For smoothing, we used a Gaussian kernel of 8mm full-width at half-maximum.

Region of interest analysis

[^{11}C]PIB binding potentials were also sampled from anatomically defined regions of interest (ROIs). The selection of ROIs was independent of the voxelwise results and similar to our previous work[17]. The MAPER (multi-atlas propagation with enhanced registration) procedure was used to generate ROIs in native space[18, 19]. This approach minimises the potential sampling error associated with misregistration and brain atrophy. The procedure has previously been shown to yield accurate and robust segmentations in normal subjects and patients with dementia.

The following regions were chosen to provide a representative sampling of cortical and sub-cortical structures and to include a number of regions likely to be affected by TBI: the anterior and posterior cingulate cortices, inferior and superior frontal gyri, occipital lobes, hippocampi, cerebellum, caudate, putamen, and thalamus. Bilateral masks were combined into a single ROI.

To improve sampling accuracy, ROI masks were intersected with thresholded tissue probability maps determined from MRI. As in [20], a 90% threshold (i.e. >0.9) was applied to the GM and WM probability maps to create tissue-specific masks. The cortical and cerebellar ROIs were then intersected with the GM tissue mask, and subcortical regions with the union of GM and WM masks.

To confirm that the hippocampal ROI results were not an effect of mislabeling due to atrophy, sampling was repeated on hippocampal masks that were manually segmented using a harmonized protocol[21].

Manual segmentation of lesions and sampling of [^{11}C]PIB binding in lesions

In TBI patients with focal injuries, lesions apparent on T1 imaging were manually segmented. Voxels corresponding to lesions were excluded from ROI and voxelwise analyses. In the ROI analysis, voxels which occurred within a lesion mask were excluded from calculation of the mean regional binding of ROIs. In the voxelwise analyses, the statistical contrasts incorporated a tool which uses voxelwise regressors to exclude individual (inconsistently located) lesions from the analysis.[22] A group lesion map was also created by summing the co-registered lesion masks.

We also investigated [^{11}C]PIB binding within lesions, the lesion penumbra, and normal-appearing gray matter in the same hemisphere. We defined spherical ROIs with a 4 mm radius around the most prominent lesion and in the gray matter of the superior temporal gyrus on the same side, as this region was not seen to be affected by lesions in any patients.

Diffusion tensor image acquisition

For MRI diffusion tensor imaging (DTI) in TBI patients, diffusion-weighted volumes with gradients applied in 16 non-collinear directions were collected in each of the four DTI runs, resulting in a total of 64 directions. The following parameters were used: 73 contiguous slices, slice thickness = 2 mm, field of view (FOV) 224 mm, matrix 128 × 128 (voxel size = 1.75 × 1.75 × 2 mm^3), b value = 1000, and four images with no diffusion weighting (b = 0 s/mm^2).

Diffusion tensor imaging analysis

The images were registered to the B_0 image by affine transformation. For brain extraction, BET [23] from the FMRIB Software Library (FSL) was used[24]. Fractional anisotropy (FA) maps were generated using the FSL Diffusion Toolbox [25].

Voxelwise analysis of the FA was carried out using tract-based spatial statistics (TBSS) in FSL [26]. The FA maps were skeletonized, leaving the central section of tracts, reducing the impact of partial volume effects at the edge of tracts. Image analysis using TBSS involved the following steps: A) nonlinear alignment of each individual's FA image into common FMRIB58_FA template space; B) affine transformation of the aligned images into standard MNI152 space; C) averaging of the aligned FA images to create a 4D mean FA image; D) thinning of the mean FA image to create a mean FA skeleton representing the centre of all WM tracts, and in this way reducing partial-volume confounds; and E) thresholding of the FA skeleton at FA≥0.2 to suppress areas of extremely low mean FA and exclude those with considerable inter-individual variability.

In each individual we then calculated the mean FA sampled from a number of white matter tracts defined by the Johns Hopkins University white matter tractography atlas [27-29]. The probabilistic tracts were thresholded at 95%, and voxels within the FA skeleton were sampled from each tract. We used the left and right cingulum-cingulate tracts (which project from posterior to anterior cingulate cortex) and the left and right cingulum-hippocampus tracts (which project from the posterior cingulate to the hippocampi). These tracts were chosen because they were connected to GM regions which had shown an increased [^{11}C]PIB BP_{ND}. As a control, we also sampled the left and right corticospinal tract, since this was not directly connected to these regions. We also calculated the mean FA of the entire white matter skeleton.

Statistical analyses

Voxelwise differences in BP_{ND} between groups were assessed using non-parametric permutation tests in FSL. This approach incorporated a tool which uses voxelwise regressors to exclude individual lesions from the analysis.[22] Ten thousand random permutations were calculated to create the null distribution for assessing the test statistics.

For ROI analysis, regional BP_{ND} was compared using repeated measures ANOVA with planned post-hoc comparisons performed between the three groups using independent sample t-tests, in SPSS.

Supplementary Results

Distinct distributions of [^{11}C]PIB binding in TBI and Alzheimer's disease

Analysis of variance (ANOVA) of BP_{ND} sampled from 10 ROIs in the AD and TBI groups showed a significant group-by-region interaction (F(2.227,37.851)=23.64, P<0.001, Greenhouse-Geisser correction applied). The Partial eta-squared effect size estimate was 0.582. There was significantly increased binding in ACC (t=4.841, df=17, p<0.001), PCC (t=4.653, df=17, p<0.001), IFG (t=4.712, df=17, p<0.001), SFG (t=5.792, df=17, p<0.001), OL (t=2.437, df=17, p=0.026), caudate (t=2.989, df=17, p=0.008) and putamen (t=3.339, df=13.030, p=0.005), no difference in the thalamus and decreased binding in hippocampus (t=-3.532, df=17, p=0.003) and cerebellum (t=-4.786, df=17, p<0.001). ANOVA in the AD and Control groups also showed a group-by-region interaction (F(1.996,33.934)=22.148, P<0.001,Greenhouse-Geisser correction applied). The Partial eta-squared effect size estimate was 0.566. There was significantly increased binding in ACC (t=4.688, df=17, p<0.001), PCC (t=4.872, df=17, p<0.001), IFG (t=5.001, df=17, p<0.001), SFG (t=5.349, df=17, p<0.001), OL (t=3.479, df=17, p=0.003), caudate (t=3.202, df=17, p=0.005) and putamen (t=4.984 df=10.720, p<0.001), no difference in the thalamus and decreased binding in hippocampus (t=-2.611, df=17, p=0.018) and cerebellum (t=-5.433,

df=12.640, p<0.001). All ROI results were similar when the analyses were repeated for manually drawn hippocampal segmentations.

e-References

1. Malec JF, Brown AW, Leibson CL, et al. The mayo classification system for traumatic brain injury severity. J Neurotrauma 2007;24:1417-1424.

2. McKhann G, Drachman D, Folstein M, Katzman R, Price D, Stadlan EM. Clinical diagnosis of Alzheimer's disease: report of the NINCDS-ADRDA Work Group under the auspices of Department of Health and Human Services Task Force on Alzheimer's Disease. Neurology 1984;34:939-944.

3. Klunk WE, Engler H, Nordberg A, et al. Imaging brain amyloid in Alzheimer's disease with Pittsburgh Compound-B. Ann Neurol 2004;55:306-319.

4. Delis DC, Kaplan E, Kramer JH. Delis-Kaplan Executive Function System. San Antonio: San Antonio, TX: Psychological Corporation, 2001.

5. Reitan R. The validity of the Trail Making test as an indicator of organic brain damage. Perceptual and Motor Skills 1958;8:271-276.

6. Wechsler D. Wechsler Memory Scale- Third Edition: Administration and Scoring Manual. San Antonio: TX: Psychological Corporation, 1997.

7. Baddeley AD, Emslie H, Nimmo-Smith I. Doors and People Test: A Test of Visual and Verbal Recall and Recognition. Bury-St-Edmunds: Thames Valley Test Company, 1994.

8. Wechsler D. Wechsler Test of Adult Reading. San Antonio: TX: The Psychological Corporation, 2001.

9. Wechsler D. WASI: Wechsler Abbreviated Scale of Intelligence. San Antonio: TX: The Psychological Corporation, 1999.

10. Cohen J. Statistical power analysis for the behavioral sciences: Academic press, 2013.

11. Rowe CC, Villemagne VL. Brain Amyloid Imaging. Journal of Nuclear Medicine 2011;52:1733-1740.

12. Knight WD, Okello AA, Ryan NS, et al. Carbon-11-Pittsburgh compound B positron emission tomography imaging of amyloid deposition in presenilin 1 mutation carriers. Brain 2011;134:293-300.

13. Ikoma Y, Edison P, Ramlackhansingh A, Brooks DJ, Turkheimer FE. Reference region automatic extraction in dynamic [lsqb]11C[rsqb]PIB. J Cereb Blood Flow Metab 2013.

14. Oh H, Madison C, Villeneuve S, Markley C, Jagust WJ. Association of Gray Matter Atrophy with Age, β-Amyloid, and Cognition in Aging. Cereb Cortex 2013.

15. Chetelat G, Desgranges B, Landeau B, et al. Direct voxel-based comparison between grey matter hypometabolism and atrophy in Alzheimer's disease. Brain 2008;131:60-71.

16. Villain N, Desgranges B, Viader F, et al. Relationships between hippocampal atrophy, white matter disruption, and gray matter hypometabolism in Alzheimer's disease. J Neurosci 2008;28:6174-6181.

17. Ramlackhansingh AF, Brooks DJ, Greenwood RJ, et al. Inflammation after trauma: Microglial activation and traumatic brain injury. Annals of neurology 2011;70:374-383.

18. Heckemann RA, Keihaninejad S, Aljabar P, Rueckert D, Hajnal JV, Hammers A. Improving intersubject image registration using tissue-class information benefits robustness and accuracy of multi-atlas based anatomical segmentation. NeuroImage 2010;51:221-227.

19. Heckemann RA, Hajnal JV, Aljabar P, Rueckert D, Hammers A. Automatic anatomical brain MRI segmentation combining label propagation and decision fusion. Neuroimage 2006;33:115-126.

20. Ikoma Y, Edison P, Ramlackhansingh A, Brooks DJ, Turkheimer FE. Reference region automatic extraction in dynamic [(11)C]PIB. J Cereb Blood Flow Metab 2013;33:1725-1731.

21. Frisoni GB, Jack CR. Harmonization of magnetic resonance-based manual hippocampal segmentation: a mandatory step for wide clinical use. Alzheimer's & dementia : the journal of the Alzheimer's Association 2011;7:171-174.

22. Blumbergs PC, Jones NR, North JB. Diffuse axonal injury in head trauma. Journal of Neurology, Neurosurgery & Psychiatry 1989;52:838-841.

23. Smith SM. Fast robust automated brain extraction. Hum Brain Mapp 2002;17:143-155.

24. Smith SM, Jenkinson M, Woolrich MW, et al. Advances in functional and structural MR image analysis and implementation as FSL. NeuroImage 2004;23 Suppl 1:S208-219.

25. Behrens TE, Johansen-Berg H, Woolrich MW, et al. Non-invasive mapping of connections between human thalamus and cortex using diffusion imaging. Nat Neurosci 2003;6:750-757.

26. Smith SM, Jenkinson M, Johansen-Berg H, et al. Tract-based spatial statistics: voxelwise analysis of multi-subject diffusion data. NeuroImage 2006;31:1487-1505.

27. Mori S, Oishi K, Jiang H, et al. Stereotaxic white matter atlas based on diffusion tensor imaging in an ICBM template. Neuroimage 2008;40:570-582.

28. Hua K, Zhang J, Wakana S, et al. Tract probability maps in stereotaxic spaces: analyses of white matter anatomy and tract-specific quantification. NeuroImage 2008;39:336-347.

29. Wakana S, Jiang H, Nagae-Poetscher LM, van Zijl PC, Mori S. Fiber tract-based atlas of human white matter anatomy. Radiology 2004;230:77-87.
