# Supplementary material for: Amyloid pathology and axonal injury after brain trauma
Source: Neurology. 2016 Mar 1;86(9):821–8. doi: 10.1212/WNL.0000000000002413 (PMC4793784; doi:10.1212/WNL.0000000000002413)
Supplement: Data Supplement [file supp_WNL.0000000000002413_Table_e-1.pdf]

Table e-1. Neuropsychological test results: traumatic brain injury (TBI) and Controls

Cognitive Domain	Cognitive Variable	TBI Mean±SD	Control Mean±SD	TBI vs. Control	Cohen's d
**Processing speed**					
Visual search	TMT Trail A (s)	32.9±15.4 (*N*=9)	19.7±4.5‡ (*N*=14)	*t* = 2.50 *†	1.30
Complex	TMT Trails B (s)	60.4±28.5 (*N*=9)	43.0±12.6‡ (*N*=14)	*t* = 1.73 ns	0.86
Naming	Stroop Color Naming (s)	38.6±12.6 (*N*=8)	23.3±9.9 (*N*=14)	*t* = 3.18 **†	1.41
Reading	Stroop Word Reading (s)	26.8±6.6 (*N*=8)	28.9±6.3 (*N*=15)	*t* = − 0.78 ns	-0.34
Complex	Stroop Inhibition (s)	73.1±30.9 (*N*=8)	23.3±4.4‡ (*N*=15)	*t* = 4.53 **††	2.74
Choice reaction time	CRT median RT (ms)	459±66 (*N*=8)	408±51 (*N*=15)	*t* = 2.05 ns	0.91
**Executive function**					
Inhibition	Inhibition – naming (s)	34.5±22.3 (*N*=8)	28.7±17.6 (*N*=15)	*t* = 0.69 ns	0.30
Cognitive flexibility	Inhibition/switching – naming + reading ÷ 2 (s)	50.1±27.8 (*N*=8)	32.2±10.5 (*N*=14)	*t* = 2.18 *†	0.97
Word generation fluency	Letter Fluency F+A+S total	40.4±8.1 (*N*=8)	46.0±12.0 (*N*=15)	*t* = -1.19 ns	-0.52
Set-shifting	Alternating-switch cost TMT Trails B minus A (s)	27.7±17.7 (*N*=9)	23.3±9.8 (*N*=14)	*t*= 0.74 ns	0.32
**Memory**					
Immediate/Working	Digit Span forward	10.4±1.6 (*N*=9)	11.5±2.2 (*N*=15)	*t* = − 1.27 ns	-0.54
	Digit Span backward	6.8±1.1 (*N*=9)	7.5±2.0‡ (*N*=15)	*t* = − 0.94 ns	-0.34

Logical	WMS-III LM I first recall total	28.6±4.0 (*N*=8)	26.4±9.0‡ (*N*=15)	*t* = 0.82 ns	0.29
	WMS-III LM I total	47.3±5.7 (*N*=8)	42.7±13.7‡ (*N*=15)	*t* = 1.12 ns	0.39
	WMS-III LM II delayed	31.6±5.4 (*N*=8)	25.6±9.2 (*N*=15)	*t* = 1.69 ns	0.74
Associative	PT immediate recall total	25.5±5.9 (*N*=9)	26.9±7.1 (*N*=15)	*t* = −0.49 ns	-0.20
**Intellectual ability**					
Reading	WTAR age-scaled score	107±10.8 (*N*=8)	112±9.8 (*N*=15)	*t* = − 1.11 ns	-0.49
Verbal	WASI Similarities	39.3±3.9 (*N*=8)	34.5±6.3‡ (*N*=15)	*t* = 2.25 *	0.85
Non-verbal	WASI Matrix Reasoning	27.9±3.7 (*N*=9)	26.8±5.0 (*N*=15)	*t* = 0.56 ns	0.24

Neuropsychological results for TBI patient and age-matched control group. Differences in variances between patient and control group (Levene's test for equality of variances P < 0.05) shown by ‡. Significant group differences shown by * t-test $P \leq 0.05$ and **t-test $P \leq 0.01$; † Mann-Whitney U $P \leq 0.05$ and †† Mann-Whitney $P \leq 0.01$; ns indicates a non-significant result on t-test and Mann-Whitney U test. TMT = Trail Making Test; Colour Naming, Word Reading, Inhibition, Inhibition/switching, and Letter Fluency are subtests of the Delis-Kaplan Executive Function System; CRT = Choice-Reaction Task; WMS-III = Wechsler Memory Scale- Third Edition; PT = People Test; WTAR = Wechsler Test of Adult Reading; WASI = Wechsler Abbreviated Scale of Intelligence. One patient could not be assessed fully because of an upper limb disability and difficulty communicating verbally.
