# Supplementary material for: Amyloid pathology and axonal injury after brain trauma
Source: Neurology. 2016 Mar 1;86(9):821–8. doi: 10.1212/WNL.0000000000002413 (PMC4793784; doi:10.1212/WNL.0000000000002413)
Supplement: Accompanying Editorial [file supp_86_9_821_v2_index.html]

Accompanying Editorial 

# Amyloid pathology and axonal injury after brain trauma

## Accompanying Editorial

**Neurology® data supplements are not copyedited before publication. Published editorials and translations have been copyedited.  
 © 2016 American Academy of Neurology.  
  
 Files in this Data Supplement:**

- Accompanying Editorial - PDF file
