# Supplementary material for: Amyloid pathology and axonal injury after brain trauma
Source: Neurology. 2016 Mar 1;86(9):821–8. doi: 10.1212/WNL.0000000000002413 (PMC4793784; doi:10.1212/WNL.0000000000002413)
Supplement: Accompanying Editorial [file supp_WNL.0000000000002413_798.pdf]

EDITORIAL

# Amyloid plaques in TBI

## Incidental finding or precursor for what is to come?

Ansgar J. Furst, PhD
Erin D. Bigler, PhD

Correspondence to
Dr. Furst:
ajfurst@stanford.edu

*Neurology*® 2016;86:798–799

Over the past decade, the rate of traumatic brain injury (TBI)-related emergency department visits has increased by 70% and was estimated in 2010 at a staggering 2.5 million visits. This number is an underestimate as it does not include individuals who did not seek/receive medical care, are part of the US military service, or cared for by the Department of Veterans Affairs. One estimate states that 3 to 5 million Americans live with a TBI-related disability.[1] Of particular concern, recent data suggest an increased risk of dementia after TBI. However, it remains unclear whether there is indeed a specific link between the occurrence of Alzheimer disease (AD) later in life and a history of TBI. The suspicion of a causal relationship between TBI and AD mostly stems from the sudden emergence of the toxic β-amyloid (Aβ) protein observed in the brains of even young patients. This finding has been puzzling, as Aβ is a hallmark of AD, a late-life dementia. Whether the primarily axonal increase of Aβ is simply an epiphenomenon of TBI pathology or actually a precursor to the distinctively different extraneuronal fibrillar Aβ plaques seen in AD remains elusive.

In this issue of *Neurology*®, Scott et al.[2] present evidence of amyloid plaques in the brains of nonsymptomatic patients with TBI who are in their mid-40s. The study compared a small group of patients with chronic (time since injury >11 months) moderate–severe TBI with a group of patients with probable AD and some controls. The finding of Aβ plaques in younger patients with chronic TBI had been recently reported by 2 other groups.[3,4] However, the novelty of the Scott et al. study lies in that they first directly compared the spatial distribution and extent of amyloid pathology among AD, TBI, and controls using the gold-standard amyloid PET tracer, Pittsburgh compound B (PiB); but then, in an important next logical step, related the location and amount of cerebral amyloid with white matter damage measured through MRI (diffusion tensor imaging) in the same patients. This is a rare study relating the signature axonal injuries seen in TBI with the curious co-occurrence of Aβ plaques. Of note, although the spatial distribution of plaques in TBI and AD was mostly frontotemporoparietal, the cerebellum had an increased amyloid burden in TBI only. The latter is noteworthy as in AD this part of the brain is typically unaffected by Aβ plaques even at late stages of the disease. A potentially specific cerebellar amyloidosis in TBI is intriguing as there have been reports of marked glucose hypometabolism[5] and cerebellar atrophy in such patients including children.[6,7]

Scott and colleagues also report elevated levels of Aβ burden in the precuneus/posterior cingulate cortex (PCC) of TBI compared to controls, which is most interesting, as this region is known to become hypometabolic in very early presymptomatic stages of AD. However, arguably the most important finding from the study is that increasing degrees of amyloid burden in this area were further associated with more severe damage in the cingulum bundle fiber tracts in TBI only. The PCC amyloid burden increased as a function of time elapsed since injury. The latter finding together with the fact that the PCC and the cingulum bundle are anatomically connected led the authors to the thought-provoking suggestion of a mechanistic link between the early TBI-associated axonal injury in the cingulum and the later spread of amyloid plaques in the adjacent PCC. Scott et al. speculate that the Aβ accumulated immediately after injury is slowly (presumably over years) spreading from neuron to neuron via a prion-like transsynaptic mechanism to the PCC. The possibility of such a disease mechanism is certainly thought-provoking, but would need to be confirmed using longitudinal data (the study by Scott et al. was only cross-sectional). Although exciting, the results from the study should be generally treated as preliminary. Only a small sample of 9 patients with TBI was examined and TBI pathology was heterogeneous: posttraumatic amnesia duration was anywhere from 2 hours to >6 months, coupled with either no focal lesion, or as severe an injury as a parietotemporal lobectomy in one patient. However, given the

See page 821

From the Departments of Psychiatry and Behavioral Sciences (A.J.F.) and Neurology and Neurological Sciences (A.J.F.), Stanford University School of Medicine, Stanford, CA; War Related Illness and Injury Study Center (WRIISC) (A.J.F.), VA Palo Alto Health Care System, Palo Alto, CA; and Department of Psychology (E.D.B.), Brigham Young University, Provo, UT.

Go to Neurology.org for full disclosures. Funding information and disclosures deemed relevant by the authors, if any, are provided at the end of the editorial.


© 2016 American Academy of Neurology

© 2016 American Academy of Neurology. Unauthorized reproduction of this article is prohibited.

inherent heterogeneity of TBI, controlling for these factors is difficult.

Scott et al. nicely demonstrate the combined use of neuroimaging biomarkers for the study of different pathologies across different diagnostic groups. It is unclear what their findings mean for the clinic however. The increased PiB uptake in patients with TBI was not associated with increased cognitive impairment, which is probably expected given the young age of the patients. It remains to be seen whether the increased PiB uptake in young chronic TBI, especially at the mild end of the TBI spectrum, does indeed constitute very early signs of AD pathology or whether Aβ plaques mean something completely different in this context. The differential cerebellar uptake would suggest a different pathology. Although Scott et al. offer an interesting explanation concerning the potential spread of Aβ, it may turn out to be too simplistic. For example, why would amyloid travel only to the PCC but not the entorhinal cortex/hippocampus despite the cingulum having many projections to this area? Future studies should also consider genetic risk factors for AD, such as *APOE* carrier status, as it moderates amyloid accumulation and metabolic decline. Similarly, the premorbid cognitive reserve afforded to an individual with TBI is likely to affect disease outcome.[8] Given that a large percentage of patients with chronic TBI have sleep/wake disturbances, the effect of sleep on amyloid turnover should also be considered in this population.[9,10]

## STUDY FUNDING

No targeted funding reported.

## DISCLOSURE

The authors report no disclosures relevant to the manuscript. Go to Neurology.org for full disclosures.

## REFERENCES

1. CDC. CDC|TBI Data and Statistics|Traumatic Brain Injury|Injury Center [online]. Available at: http://www.cdc.gov/traumaticbraininjury/data/index.html. Accessed November 2015.
2. Scott G, Ramlackhansingh AF, Edison P, et al. Amyloid pathology and axonal injury after brain trauma. Neurology 2016;86:821–828.
3. Hong YT, Veenith T, Dewar D, et al. Amyloid imaging with carbon 11-labeled Pittsburgh compound B for traumatic brain injury. JAMA Neurol 2014;71:23–31.
4. Yang ST, Hsiao IT, Hsieh CJ, et al. Accumulation of amyloid in cognitive impairment after mild traumatic brain injury. J Neurol Sci 2015;349:99–104.
5. Peskind ER, Petrie EC, Cross DJ, et al. Cerebrocerebellar hypometabolism associated with repetitive blast exposure mild traumatic brain injury in 12 Iraq war Veterans with persistent post-concussive symptoms. Neuroimage 2011;54(suppl 1):S76–S82.
6. Kim J, Avants B, Patel S, et al. Structural consequences of diffuse traumatic brain injury: a large deformation tensor-based morphometry study. Neuroimage 2008;39:1014–1026.
7. Spanos GK, Wilde EA, Bigler ED, et al. cerebellar atrophy after moderate-to-severe pediatric traumatic brain injury. AJNR Am J Neuroradiol 2007;28:537–542.
8. Bigler ED, Stern Y. Traumatic brain injury and reserve. Handb Clin Neurol 2015;128:691–710.
9. Mander BA, Marks SM, Vogel JW, et al. Beta-amyloid disrupts human NREM slow waves and related hippocampus-dependent memory consolidation. Nat Neurosci 2015;18:1051–1057.
10. Ouellet MC, Beaulieu-Bonneau S, Morin CM. Sleep-wake disturbances after traumatic brain injury. Lancet Neurol 2015;14:746–757.



© 2016 American Academy of Neurology. Unauthorized reproduction of this article is prohibited.
